# Confirmation of human protein interaction data by human expression data

**DOI:** 10.1186/1471-2105-6-112

**Published:** 2005-05-06

**Authors:** Andreas Hahn, Jörg Rahnenführer, Priti Talwar, Thomas Lengauer

**Affiliations:** 1 Max-Planck-Institute for Informatics, Stuhlsatzenhausweg 85, D-66123 Saarbrücken, Germany

## Abstract

**Background:**

With microarray technology the expression of thousands of genes can be measured simultaneously. It is well known that the expression levels of genes of interacting proteins are correlated significantly more strongly in *Saccharomyces cerevisiae *than those of proteins that are not interacting. The objective of this work is to investigate whether this observation extends to the human genome.

**Results:**

We investigated the quantitative relationship between expression levels of genes encoding interacting proteins and genes encoding random protein pairs. Therefore we studied 1369 interacting human protein pairs and human gene expression levels of 155 arrays. We were able to establish a statistically significantly higher correlation between the expression levels of genes whose proteins interact compared to random protein pairs. Additionally we were able to provide evidence that genes encoding proteins belonging to the same GO-class show correlated expression levels.

**Conclusion:**

This finding is concurrent with the naive hypothesis that the scales of production of interacting proteins are linked because an efficient interaction demands that involved proteins are available to some degree. The goal of further research in this field will be to understand the biological mechanisms behind this observation.

## Background

Gene expression data [1-3] and protein interaction data [4] are two types of data produced in the bioinformatics field. We investigated whether human gene expression levels of interacting protein pairs show a higher degree of dependence than those of random protein pairs. To date, such studies have only been performed in lower organisms like *S. cerevisiae *[5,6], in a comparative study using bacteriophage T7 and *S. cerevisiae *[7], and in *C. elegans *[8].

The first global evidence that genes with similar expression profiles are likely to encode interacting proteins has been provided in a study on *S. cerevisiae *by Ge et al. [5]. They compared the probability of interaction between proteins encoded by genes that belong to common expression profiling clusters with the probability of interaction between proteins encoded by genes that belong to different clusters. They found that proteins from the intra-group genes are more than five times as likely to interact with each other as proteins from the inter-group genes.

Tornow et al. [6] used superparamagnetic clustering to integrate protein interaction and expression data from independent experiments in *S. cerevisiae *and revealed hypothetical functional protein modules. Grigoriev [7] demonstrated the similarity of expression patterns for a pair of genes and interaction of the proteins they encode for both the bacteriophage T7 and in *S. cerevisiae*. He found the mean correlation coefficients of gene expression profiles between interacting proteins to be significantly higher than those between random protein pairs.

Recently Li et al. [8] analysed the transcriptome and interactome data of *C. elegans *and discovered that the correlation is lower than expected from observations in yeast.

A study by Jansen et al. [9] links gene expression on a genomic scale with protein-protein interaction in *S. cerevisiae*. They showed that while the subunits of the permanent protein complexes do indeed share significant correlation in their RNA expression, the correlated expression is relatively poor in detecting transient interactions. In a comprehensive study about *S. cerevisiae *conducted by Kemmeren et al. [10], up to 71% of the biologically verified interactions could be validated with the gene co-expression approach. Integration of expression and interaction data is thus a way to improve the confidence of protein-protein interaction data generated by high-throughput technologies. Kemmeren et al. [11] see enormous challenges in large genomes (orders of magnitude larger than *S. cerevisiae*) because of poor annotation, non-standardised gene names, and more complex interactions with the environment.

## Results

### Expression levels of genes encoding interacting proteins are correlated more strongly

**Figure 1:**
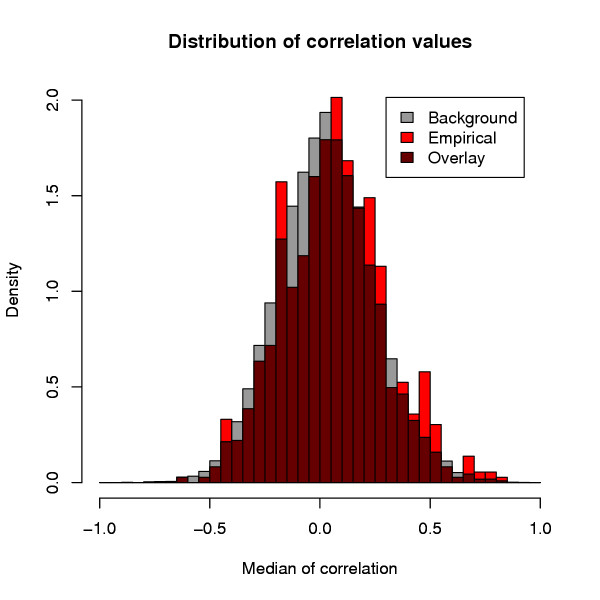
**Empirical and background distribution of correlation values**. For each interaction pair and for each dataset we calculated the correlation of expression levels of genes encoding interacting protein pairs. The graph shows slightly higher correlation values in the datasets (empirical distribution) than the correlation in the case of random protein pairs (background distribution).

Using five publicly available human expression datasets (Table 1) and 1369 human interacting protein pairs we compared the correlation of expression of genes encoding interacting proteins (empirical distribution) with the correlation of random protein pairs (background distribution). Figure 1 shows that the distribution of empirical correlations is slightly shifted to the right compared to the distribution of correlations in the case of random protein pairs. This result implies that in our data interacting proteins are preferentially encoded by coregulated genes.

**Figure 2:**
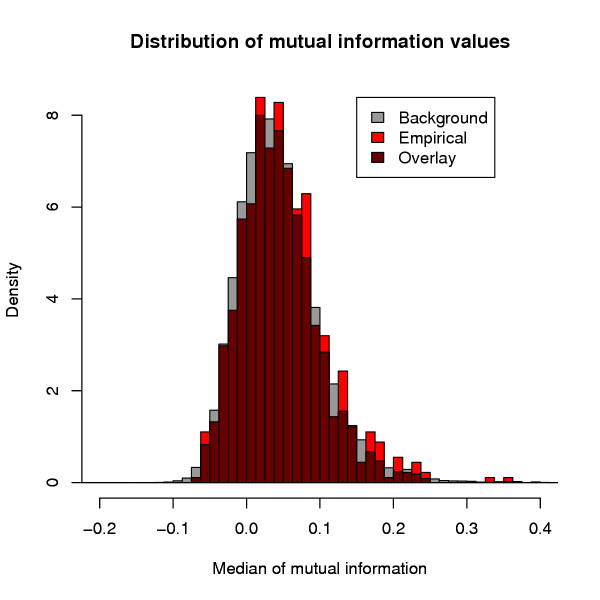
**Distribution of mutual information**. For each interaction pair and for each dataset we calculated the mutual information of expression levels of genes encoding interacting protein pairs. The graph shows empirical and background distribution to be very similar.

Using mutual information as a measure of dependence the shift observed in figure 1 has almost vanished (figure 2). The difference between empirical and background distribution of the medians is 0.04 in the correlation case and <0.01 in the mutual information case. This observation suggests that correlation as a measure of dependence is more suitable than mutual information when analyzing dependencies between expression levels of interacting proteins. In the Methods section we give a possible interpretation of this observation.

Using each dataset separately we tested the hypothesis that correlation between expression of genes encoding *interacting *protein pairs is not higher than correlation between expression of genes encoding *random *protein pairs. The detailed algorithms are given in the Methods section. For four out of five analysed datasets this hypothesis is rejected at a significance level *α *= 0.05. This means that in these four datasets the correlation of expression levels of genes which encode interacting proteins is statistically significantly higher than the correlation of expression levels in genes which encode random pairs of proteins.

### Increased p-values by use of mutual information instead of correlation

Using mutual information instead of correlation as a measure of dependence between gene expression levels leads to increased p-values for each of the five datasets. Thus the significance results of the analysis with correlation as dependence measure do not hold when using mutual information as dependence measure. This may be caused by the fact that most dependencies between expression levels are linear or close to linear and not parabolic which would preferentially be discovered by the mutual information measure. Because the correlation coefficient seems to be the more appropriate measure of dependence for this analysis we do not discuss mutual information in the following.

### Expression of genes involved in different biological processes

**Figure 3:**
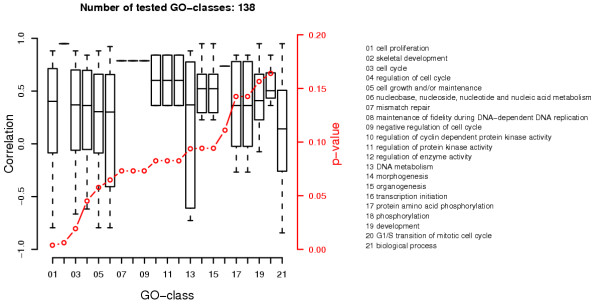
**Correlations and p-values of the expression dataset from Chi**. The diagram contains the contains the box-and-whisker plots and the p-values of the twenty GO-classes that yield the most significant results for the respective dataset and of the GO-class *biological process*. It shows for different GO-classes, how strongly the expression levels of genes that encode interacting proteins from this common GO-class are correlated. The GO-classes along the x-axis are ordered by the corresponding p-value. This p-value gives the probability to get the depicted correlation results using random interacting protein pairs from the respective GO-class. For comparison the GO-class 'biological process', which comprises all interaction pairs (except the self-interactions), has been added.

**Figure 4:**
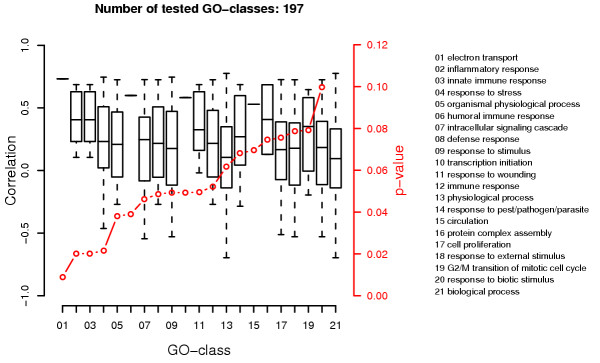
**Correlations and p-values of the expression dataset from Higgins**. Analogous to figure 3

**Figure 5:**
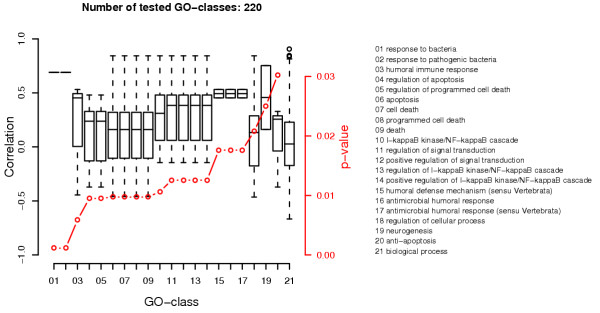
**Correlations and p-values of the expression dataset from Pathan**. Analogous to figure 3

**Figure 6:**
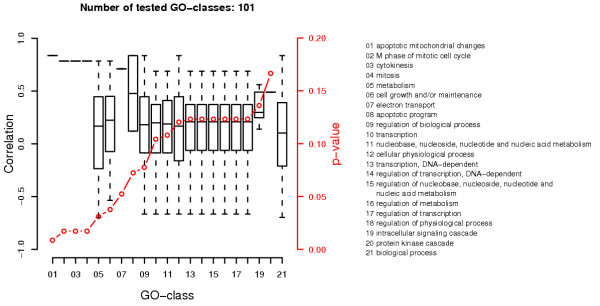
**Correlations and p-values of the expression dataset from Zhang**. Analogous to figure 3

**Figure 7:**
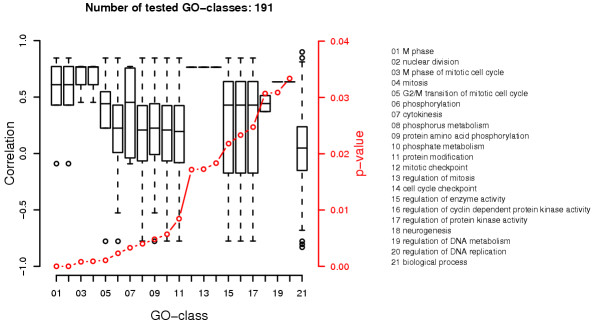
**Correlations and p-values of the expression dataset from Zhao**. Analogous to figure 3

Assigning GO-classes to the 1369 protein interactions as described in the Methods section below, for each dataset we analysed the expression levels of genes encoding interacting protein pairs both belonging to the same GO-class. Each of the figures 3, 4, 5, 6, 7 contains the box-and-whisker plots and the p-values of the twenty GO-classes that yield the most significant results for the respective dataset and of the GO-class *biological process*. Our method is different from the methods used by the authors that generated the datasets that we analysed. Thus our results cannot be compared directly with theirs. However, we feel that it is still useful to point out the similarity of some of our findings with the observations of these authors.

In the following we refer to the different datasets by the number they have received in table 1.

Figure 3 shows that in dataset 1 mainly genes included in the GO-classes *cell cycle*, *cell growth*, and *cell proliferation *are highly correlated. All in all only few GO-classes show low p-values which, in principle, agrees with the observations of Chi et al. [12] that siRNA-mediated gene silencing leads to only small variations in the gene expression pattern.

In figure 4, describing our results of the second expression dataset of Higgins et al. [13], many genes included in *immune response *and *inflammatory response *like interleukins, chemokine receptors, and chemokine ligands are highly correlated. This is an indication that the expression of chemokines and chemokine receptors is spatially and temporally restricted not only in the developing human kidney as reported in Gröne et al. [14] but also in the fully developed kidney.

Figure 5 shows our results of the GO-expression analysis of the third dataset. In this dataset Pathan et al. [15] found genes that are involved in bacterial infection to be significantly upregulated in blood after exposure to meningococci. We found the expression of genes that are involved in the *response to (pathogenic) bacteria *to be highly correlated which is in concurrence with the findings of Pathan et al. [15].

Zhang et al. [16] analysed the changes in transcript abundance occurring during senescence in human fibroblasts, as compared with early passage proliferating cells or quiescent cells. Figure 6 shows the results of our analysis of their expression dataset. In agreement with their findings we observed a strong correlation of genes that relate to *apoptosis *and genes that relate to *transcription*, but in contrast to them, we could not find significant correlation of genes that are involved in the *cell cycle regulation*.

Zhao et al. [17] analysed the effects of methylseleninic acid on the transcriptional program of human prostate cancer cells. Corresponding to their observation of decreased expression of genes involved in all phases of the *cell cycle *lines that do not express androgen receptor protein we found significant correlation of genes involved in *M phase*, *nuclear division*, and *mitosis*. Figure 7 displays the correlations and p-values of the top twenty GO-classes we calculated for the fifth dataset. In consensus with the expectations genes encoding proteins that are involved in the *cell cycle *show the lowest p-values in our analysis.

## Discussion

This study investigates the relationship between two biological phenomena – gene expression and protein-protein interaction in *H. sapiens *– based on experimental data available in public databases. The study was prompted by the fact that in yeast and other lower non-mammalian organisms correlation is observed between expression levels of genes encoding interacting proteins. We were able to obtain convincing evidence of correlation using the Pearson's correlation coefficient but could not confirm these results when taking the mutual information as a measure of dependence. Using information on the GO-class to which both proteins of an interacting protein pair belong, we were able to find significant correlations of expression levels mostly in accordance to existing knowledge.

The results of our investigation lend additional credibility to the protein-protein interaction data used.

Once more interaction data are available, an analysis of the type presented here should be repeated including information on domains and phenotypes. For instance, one of the remaining open questions is whether the correlation of expression vectors of genes encoding interacting proteins with certain compared to random combinations of domains is statistically significantly different.

Larger interaction datasets will also provide the opportunity to analyse the question if genes encoding interacting proteins are located on the same chromosome or even in close neighbourhood to each other more often than expected when assuming a random order. This problem has been addressed recently by Hurst et al. [18].

## Conclusion

In this study we observed a statistically significant correlation between expression of genes encoding interacting proteins in *H. sapiens*. This finding points towards a biological mechanism which coregulates the expression of such genes. Additionally it confirms the relevance of using gene expression data and interaction data in human genome analysis.

## Methods

### Gene expression data

For our study we used public datasets from the Stanford Microarray Database (SMD) [19]. This database includes much actual expression data from the same (cDNA microarray) platform, which is an important prerequisite for a well-founded analysis [20]. Datasets were selected by the following criteria:

• At least 20 000 clones per array

• At least 20 arrays per dataset

• Equal sets of measured clones per dataset

• Publication not earlier than 2003.

The following datasets were included in our study: Chi et al. [12] (human kidney cells), Higgins et al. [13] (normal tissue of kidney), Pathan et al. [15] (infection of blood cells), Zhang et al. [16] (gene transcription occurring during replicative senescence in human fibroblasts and mammary epithelial cells), and Zhao et al. [17] (effects of methylseleninic acid on the transcriptional program of prostate cancer LNCaP (Lymph Node Carcinoma of the Prostate) cells). The number of arrays ranges from 21 to 42 and the number of measured clones from 31 736 to 43 196. As expression level we used the binary logarithm of the normalised ratio of gene signal (channel 2) and reference signal (channel 1).

### Protein-protein interaction data

As protein interaction database we used DIP [21] listing protein pairs that are known to interact with each other, because DIP allows the user to select interactions based on their species of origin (e.g. human). Interaction here means that two amino acid chains were experimentally identified to bind to each other. In September 2004 the database comprised 1369 human protein-protein interaction pairs including 51 self-interactions. These self-interactions were excluded from the analysis because the corresponding gene expression levels (which are two identical vectors) always have correlation 1.

### Matching gene and protein identifiers

In order to determine the expression levels of genes encoding proteins that interact we have to know which proteins are encoded by which genes. Thus we have to match gene identifiers with protein identifiers. Specifically, we matched UniGene cluster IDs [22] from the expression files of the SMD [19] with Swiss-Prot accession numbers [23] (e.g. sp:Q07812), with PIR accession numbers [24] (e.g. pir:A47538), and with NCBI sequence identification numbers [25] (e.g. gi:539664) of the DIP files. As 'translator' we used a file called 'Hs.data' from the NCBI website [26] which contains the mentioned identifiers and the corresponding UniGene cluster IDs. In order to limit runtime, we refrained from applying sophisticated selection methods [27] where multiple matching occurred, but considered the first hit at all times. By using this approach, for 87% of the interacting protein pairs the genes encoding these proteins can be determined. In cases where this procedure was not successful, we used information from the Harvester website [28]. By this combination of methods the proportion increases from 87% to 94%. For many of these protein interactions no expression data of the encoding genes are available. Depending on the number of genes measured in the five expression data sets for at least 43% (dataset 4) and for up to 72% (dataset 3) of the proteins the corresponding gene expression levels can be determined. For evaluating the amount of dependence between the expression levels of two genes encoding interacting proteins, the expression of both genes has to be measured. Disregarding self-interactions this is the case in at least 10% (dataset 4) and up to 47% (dataset 3) out of 1369 interactions.

### Pearson's correlation coefficient

Let (*X*_1_, *Y*_1_), (*X*_2_, *Y*_2_),..., (*X_n_*, *Y_n_*) be the *n *pairs of expression levels of two random variables *X *(expression of first protein) and *Y *(expression of second protein). We wish to measure the degree to which *X *and *Y *are linearly dependent as opposed to being independent. The correlation then is defined by



### Mutual information

Mutual information measures the mutual dependence of two variables based on information theory.

Two random variables, *X *and *Y*, with probability distributions *p*_*X*_(*x*) and *p*_*Y*_(*y*) and the joint distribution *p*_*XY*_(*x*, *y*) are statistically independent if

*p*_*XY*_(*x*, *y*) = *p*_*X*_(*x*)·*p*_*Y*_(*y*).     (2)

The mutual information



quantifies the degree of dependence of *X *and *Y *using the distance between the joint distribution and the distribution in case of total independence. The mutual information becomes large if *X *and *Y *contain the same information.

### Calculating the degree of dependence between expression levels of genes encoding interacting proteins for all datasets

In our analysis we used expression vectors each containing the expression levels of one gene from all arrays of a dataset extracted from the SMD [19]. For each dataset we determined the correlation of vectors each containing the expression levels of genes encoding two interacting proteins. For each interaction the median of the resulting set of five (one for each dataset) correlation coefficients was calculated. We used a permutation approach (with 10 000 permutations) to compare the empirical correlation and mutual information with the corresponding background distributions. In each permutation step we held the expression levels *X *of one protein fixed and permuted the interaction partners encoded by genes with expression levels *Y*. Thus for each permutation we got a new interaction dataset with random protein pairs. For each of these datasets we calculated the correlation values and their median as before for the original dataset.

We repeated this procedure using mutual information as measure of dependence. The distributions of correlation and mutual information are shown in figure 1 and in figure 2, respectively.

### Calculating p-values for each dataset

As before we determined the correlation of vectors for each dataset, each vector containing the expression levels of genes encoding two interacting proteins. We also calculated these values for the permuted datasets (1000 permutations). To get more specific results, we did not use the median of the correlations or mutual information values here but performed the permutation approach for each dataset separately.

Denote with *n*_*perm *_the number of permutations and with *n*_*high *_the number of correlation and mutual information values higher than those in the original dataset. Then the estimated p-values are given by

*p *= *n*_*high*_/*n*_*perm*_.     (5)

The corresponding p-values are shown in table 2.

### GO analysis in each dataset

To further elucidate dependencies between expression levels in the five datasets we analysed for each dataset if genes encoding proteins within different GO-classes representing biological processes have correlated expression levels. Therefore, using QuickGO [29] we determined for each GO-class describing a biological process which of the 1369 interacting pairs include proteins, both of which belong to the respective biological process. We used these sets of interacting protein pairs to find biological processes that include protein pairs encoded by genes with highly correlated gene expression levels. By the use of a permutation test we compared the correlations of protein pairs belonging to a certain GO-class with the correlations of protein pairs not belonging to that GO-class. Analogous to the case without differentiation between GO-classes we can apply equation (5) again to get a p-value for each GO-class in each dataset (figure 3, 4, 5, 6, 7). We did not perform a correction procedure for multiple testing because the tested GO-classes often include very similar or even identical sets of interactions. Essential in this analysis is the ranking of the GO-classes.

## Authors' contributions

AH developed and implemented the method, ran the calculations and prepared a draft of the paper. The other authors contributed to the development of the method, the interpretation of the results, and the refinement of the paper.

## Figures and Tables

**Table 1:** Information on expression datasets. The study includes a total of 155 arrays from five datasets. Each dataset has been published not earlier than 2003 and includes at least 20 arrays and 30 000 spots.

Number of dataset	Dataset	Year	Number of arrays	Number of spots
1	Chi [12]	2003	27	43 196
2	Higgins [13]	2004	34	43 196
3	Pathan [15]	2004	42	37 632
4	Zhang [16]	2003	21	31 736
5	Zhao [17]	2004	31	43 196

**Table 2:** P-values using correlation and mutual information as measure of dependence. The p-values describe the probability of obtaining a higher correlation (third column) or mutual information (fourth column) than the one observed, assuming that the expression levels of genes encoding interacting proteins are independent. The values have been estimated using 1000 permutations.

Number of dataset	Dataset	p-value (correlation)	p-value (mutual information)
1	Chi [12]	0.067	0.243
2	Higgins [13]	0.008	0.277
3	Pathan [15]	0.004	0.150
4	Zhang [16]	0.016	0.264
5	Zhao [17]	0.019	0.368
